# Production of Neutrophil Extracellular Traps Contributes to the Pathogenesis of *Francisella* tularemia

**DOI:** 10.3389/fimmu.2020.00679

**Published:** 2020-04-24

**Authors:** Sivasami Pulavendran, Maram Prasanthi, Akhilesh Ramachandran, Rezabek Grant, Timothy A. Snider, Vincent T. K. Chow, Jerry R. Malayer, Narasaraju Teluguakula

**Affiliations:** ^1^College of Veterinary Medicine, Oklahoma State University, Stillwater, Oklahoma, OK, United States; ^2^Department of Microbiology and Immunology, School of Medicine, National University of Singapore, National University Health System, Singapore, Singapore

**Keywords:** *Francisella*, neutrophil extracellular traps, myeloperoxidase, NEtosis, alveolar injury

## Abstract

*Francisella tularensis*(*Ft*) is a highly virulent, intracellular Gram-negative bacterial pathogen. Acute *Ft* infection by aerosol route causes pneumonic tularemia, characterized by nodular hemorrhagic lesions, neutrophil-predominant influx, necrotic debris, fibrin deposition, and severe alveolitis. *Ft* suppresses activity of neutrophils by impairing their respiratory burst and phagocytic activity. However, the fate of the massive numbers of neutrophils recruited to the infection site is unclear. Here, we show that *Ft* infection resulted in prominent induction of neutrophil extracellular traps (NETs) within damaged lungs of mice infected with the live attenuated vaccine strain of *Ft*(*Ft*-LVS), as well as in the lungs of domestic cats and rabbits naturally infected with *Ft*. Further, *Ft*-LVS infection increased lung myeloperoxidase (MPO) activity, which mediates histone protein degradation during NETosis and anchors chromatin scaffolds in NETs. In addition, *Ft* infection also induced expression of peptidylarginine deiminase 4, an enzyme that causes citrullination of histones during formation of NETs. The released NETs were found largely attached to the alveolar epithelium, and disrupted the thin alveolar epithelial barrier. Furthermore, *Ft* infection induced a concentration-dependent release of NETs from neutrophils *in vitro.* Pharmacological blocking of MPO reduced *Ft*-induced NETs release, whereas addition of H_2_O_2_ (a substrate of MPO) significantly augmented NETs release, thus indicating a critical role of MPO in *Ft-*induced NETs. Although immunofluorescence and electron microscopy revealed that NETs could efficiently trap *Ft* bacteria, NETs failed to exert bactericidal effects. Taken together, these findings suggest that NETs exacerbate tissue damage in pulmonary *Ft* infection, and that targeting NETosis may offer novel therapeutic interventions in alleviating *Ft*-induced tissue damage.

## Introduction

*Francisella tularensis*(*Ft*) is a zoonotic, intracellular bacterial pathogen, known to cause the disease tularemia that affects major organs including lungs, liver, and spleen (1–3). *Ft* can transmit by direct skin contact or through mucosal, respiratory or gastrointestinal tract routes. The aerosol route of *Ft* infection causes pneumonic tularemia, a severe form of the disease with high fatality rates (4–6). Several studies support that aggravated immune response with massive neutrophils influx contribute to host tissue destruction and pathophysiology in *Ft* infection (7–9). Together with necrotic debris and bacteria, excessive neutrophils recruitment clogs the bronchioles and alveolar airspaces, results in granuloma formation (7, 10–12). It has been reported that induction of matrix metalloproteinase 9 (MMP 9) aggravates neutrophil influx and mortality in *Ft* infection and that mice lacking MMP 9 gene (MMP 9 ^–/–^) exhibit diminished neutrophils recruitment and improved survival in both vaccine strain of *Ft* (*Ft*-LVS) and also highly virulent Type A strain (SchuS4) infections (13). Although these findings implicate massive neutrophil influx in the pathogenesis of *Ft* infection, the fate of these neutrophils at the infection site is unknown. Further, mechanisms of how these infiltrated neutrophils contribute to *Ft* pathophysiology remain largely unexplored.

Both neutrophils and macrophages are targets for *Ft* infection (14, 15). Several studies have demonstrated that *Ft* parasitizes inside the neutrophils and disrupts their antimicrobial defense by suppressing neutrophil respiratory burst (15, 16). One proposed mechanism is that *Ft* inhibits assembly of NADPH oxidase enzyme complex, which is required for reactive oxygen species (ROS) generation, and by inhibiting neutrophil respiratory burst, it escapes from phagosomal lysis (17). Neutrophils are terminally differentiated and short-lived cells which undergo constitutive apoptosis. *Ft* infection has been shown to prolong their lifespan by inhibiting apoptotic signaling mechanism (18). Previous *in vitro* studies on *Ft*-neutrophil interactions have implicated in implicated impaired neutrophil function *in vitro*, but the activation status of the pulmonary sequestered neutrophils is unclear. Emerging evidence supports a potential role of excessive neutrophil influx, their activation and NETosis in the pathophysiology of various bacterial and viral diseases (19–23). NETs were initially discovered for their role in bacterial killing (24). However, we found that presence of excessive NETs in *Streptococcus pneumoniae (S. pneumoniae)* superinfection following influenza infection in mice does not reduce bacterial load, but augmented bacterial burden and aggravated pathology (19). During NETs formation, the released chromatin strands are decorated with toxic proteins such as histones, MPO and neutrophil elastase (NE), which contribute to host pathology (24). We have previously reported NETs embroiled with thin alveolar-capillary surfaces of the lungs during severe influenza and *S. pneumoniae* superinfection following influenza which mediate cytotoxic effects in alveolar epithelial and endothelial cells (19, 25). Further, our recent studies have identified extracellular histones as major mediators in NETs-induced tissue damage and organ failure (23).

Although massive neutrophil influx is implicated in *Ft* pathophysiology, there are hitherto no *in vivo* reports on NETs induction or their role in tissue damage during *Ft* infection. In the present study, we characterized the induction of NETs in *Ft-*LVS infected mice. Further, accumulation of NETs was also evaluated in clinically diagnosed *Ft*-positive domestic cats and rabbits. Our findings revealed induction of extensive NETs during *Ft* infection. We also found induction of NETosis, when mouse neutrophils were infected with *Ft*-LVS *in vitro.* The NETs induction was found to be dependent on MPO activity. Although *Ft* was found trapped in NETs during infection, NETs failed to show bactericidal effects against *Francisella*. These studies indicate that NETs release contributes to the lung pathology in *Ft* infection.

## Materials and Methods

### Bacteria, Animals, and Ethics Approval

Live attenuated vaccine strain of *Francisella tularensis* LVS (*Ft*-LVS); and *Ft*-LVS-GFP (GFP labeled) American Type Culture Collection (ATCC, VA) were provided by Dr. Jerry Malayer. The bacteria were cultured on chocolate agar plates at 37°C in a 5% CO_2_incubator. For bacterial counts, a serial 10-fold dilutions were made from a single colony in phosphate buffered saline (PBS) and colony forming units (CFU) were determined. For preparation of formalin-killed bacteria, the bacteria were incubated with 4% neutral buffered formalin for 1 h at 37°C and washed with PBS to remove formalin. BALB/c mice (6–8 weeks old) were used in this study. The animals were housed in microisolator cages in a BSL-2 animal facility. All animal experiments were approved by the Institutional Animal Care and Use Committee (IACUC) of Oklahoma State University (protocol number VM-17-36) and were performed in strict accordance with their recommendations.

### Ft-Infected Clinical Samples

Severely ill domestic rabbits (*n* = 4) and cats (*n* = 3) were brought to the Oklahoma State University veterinary clinic with symptoms of high fever, diarrhea, nasal blood discharge, lethargy and respiratory complications. Animals were euthanized and performed necropsy analysis at Oklahoma Animal Disease and Diagnostic Laboratory (OADDL). Routine RT-PCR analysis on liver and spleen specimens identified *Francisella* positive infection. For histopathologic and NETs immunostaining analysis, lungs and liver tissues were fixed in neutral buffered formalin and embedded in paraffin. Uninfected rabbit lungs and liver samples (*n* = 3) were used as controls in this study.

### Mice Infections

For all infections, animals were anesthetized with a mixture of ketamine (7.5 mg/kg) and xylazine (0.1 mg/kg) by intraperitoneal injection. The mice were then intranasally challenged with a lethal dose of about 1000 CFU of *Ft*-LVS in 50 μl diluted in sterile phosphate-buffered saline (PBS). Mock-inoculated control mice received equal volumes of PBS. The actual dose of the bacteria was confirmed by plating onto chocolate-agar and CFU were determined after 72 h. *N* = 5–7 mice were used in each group. Animals were monitored for clinical signs of weight loss, lethargy, and respiratory distress.

### Histopathology Analysis

Following infection, mice were euthanized at 5 dpi, as infected animals exhibited severe clinical signs of illness with lethargy and respiratory distress. Lungs and other organs including liver, spleen, and heart were fixed in 4% formalin as described previously (23). Whole lungs from control and infected animals were fixed by the intratracheal instillation of 4% neutral-buffered formalin at 25-cm water gauge pressure and embedded in paraffin. In another set of experiments, lungs were lavaged with equal volumes of PBS as described earlier and bronchoalveolar lavage fluids were used to determine NETs release (25). A Semi-quantitative histology scores based on the lesions in the lungs, including tissue necrosis, lymphoid necrosis, Pyknotic debris, inflammation (neutrophil/macrophage/lymphocytes), alveolar damage, interstitial pneumonia, bronchopneumonia, nodular and hemorrhagic lesions, and accumulations of necrotic debris were analyzed by board certified pathologists in a blinded manner (23). For liver, scoring was based on tissue necrosis, lymphoid necrosis, Pyknotic debris, discrete/non-discrete granuloma formation and composition of degenerative, and non-degenerative neutrophil infiltrations. Histology scores were given as 0,No lesions; 1, Mild lesions; 2, Moderate lesions; and 3, Marked lesions. Similarly, pathologic analysis of lungs and liver sections from domestic *Ft*-positive cats, rabbits and uninfected rabbits were analyzed and scored in a blinded manner.

### Bacterial Burden

A portion of the lung was homogenized in sterile PBS using mortar-pestle. The homogenate was centrifuged at 100 × *g* for 10 min. A Serial 10-fold dilutions of the supernatants were prepared in sterile PBS. A 10-μl aliquots of serially diluted supernatants were plated onto chocolate-agar and quantification of the colonies was performed after 72 h incubation at 37°C as described above. The results were expressed as log_10_ CFU/milligram protein.

### Immunohistochemistry Analysis

Mock or *Ft*-infected mouse lungs and liver samples were cut into 4-μm thick section and immunohistochemistry performed to detect NET release using antibodies against citH3 and MPO as described previously (26). Briefly, lung and liver sections were deparaffinized in xylene, permeabilized with 0.5% Triton X-100, and blocked with 3% fetal bovine serum. The sections were then incubated at 4°C overnight with 1:400 dilutions of primary antibodies, i.e., mouse anti–citH3 (Abcam, MA) or anti-MPO (Abcam, MA). After washing thrice with PBS, the slides were incubated with 1:500 dilutions of secondary antibodies conjugated to Alexa Fluor 488 or Alexa Fluor 546 (Molecular Probes, Eugene, OR, United States) at room temperature for 1 h. The slides were washed thrice with PBS, mounted in medium containing DAPI (Vector Laboratories, Burlingame, CA, United States) and examined using an Olympus fluorescence microscope. Similarly, *Ft-*positive domestic cats and rabbits and control rabbits were evaluated for induction of NETs formation in their lung and liver sections by immunostaining using anti-citH3 (Abcam, MA) and monoclonal anti-MPO antibodies (Abcam, MA).

For visualizing alveolar damage during *Ft* infection, mock, and *Ft*-infected mouse lung tissue sections were stained with primary antibodies against alveolar type I epithelial membrane marker (T1α, 1: 500, DSHB, University of Iowa, IA) and citH3 (1:400, Abcam, MA) and incubated overnight at 4°C. Lung sections were then labeled with secondary Alexa Fluor antibodies as described above.

### Western Blot Analysis in the Cell-Free BAL Fluids

For collection of BAL, both mock infected and *Ft*-LVS infected mouse lungs were lavaged with equal volumes of phosphate buffered saline. The recovery of lavage fluids was more than 90% in all samples. All the western blots were carried out by loading equal volumes of BAL from control and infected animals. The samples were further centrifuged at 4000 rpm for 15 min to obtain cell-free BAL. For western blot analysis, equal volumes of BAL samples from control, infected and treatment groups were loaded, as described in our previous publications (26). The BAL fluids were analyzed for extracellular histones (H2A or H2B, 1:1000, Abcam, MA) and modified histones (citH3, 1:1000, Abcam, MA) by western blot as described previously (26). Lung injury was assessed by T1-α (1:1000, DSHB, University of Iowa). The release of peptidylarginine deiminase 4 (PAD4, 1:200, Santa Cruz, CA, United States) in the BAL was an indication of active NETosis during *Ft* infection. Densitometry on western blots carried out using Image J software (National Institutes of Health).

### Measurement MPO Activity

MPO activity in the lung homogenates from mock infected, and *Ft* infected mice was performed as described previously (17). The MPO activity was determined using the formula: units/mg protein = ΔOD/minute × 45.1, expressed as U/mg protein lung (27). One unit of the enzyme is defined as the amount that consumes 1 μmol of H_2_O_2_ per minute.

### Ft-Neutrophil Interaction Induces NETs *in vitro*

Mouse blood was collected in sodium citrate. Neutrophils were isolated as by MACS neutrophil isolation kit (Miltenyi Biotec Inc, CA) using Ly6G antibodies through a positive selection as described previously (25). The neutrophils purity was > 90%, determined by modified Giemsa staining. For *Ft*-neutrophil interaction, neutrophils were seeded at the density of 1 × 10^5^ cells onto poly-D-lysine coated 12 mm size coverslips, placed in 24 well culture plates. For determining NETs induction during *Ft*-neutrophil interaction, *Ft*-LVS was added to the neutrophils at MOI of 10:1, 20:1, 30:1, 50:1, and 100:1 diluted in DMEM medium (Gibco, Invitrogen, CA) containing 1% mouse serum, for 4 h at 37°C in a 5% CO_2_incubator. Neutrophils cultured without bacteria were used as control. To determine if viable *Ft* was required for NETs induction, neutrophils were incubated with formalin-killed *Ft*-LVS for 4 h. The NETs released were visualized using DAPI (nuclear dye) under fluorescence microscope at 400× magnification. Released NETs were also validated using NETs marker, lactoferrin (1:400, Sigma, MO) and immunocytochemistry was performed (26). We evaluated at least 5 fields on each slide to quantify the total positive cells showing NETs.

### Scanning Electron Microscopy

Mouse neutrophils, isolated as described above, were cultured on to 12-mm coverslips, infected with *Ft*-LVS (1:20), and incubated for 4 h. Cells were fixed in 2.5% glutaraldehyde for 2 h and washed in 0.2 M Cacodylate buffer three times and stored at 4°C overnight. Samples were then post-fixed for 1 h in aqueous 1% osmium tetroxide, and washed three times in Cacodylate buffer. Cells were dehydrated through a graded ethanol series (50, 60, 70, 90, and 95%) and washed three times with absolute ethanol. Finally the samples were chemically dehydrated using hexamethyldisilazane, and air-dried. Dried cells were mounted onto aluminum stubs, grounded with silver paint, and then sputter coated with 3.5 nm gold. Samples were viewed in a FEI Quanta 600 FEG scanning electron microscope. For positive release of NETs, we stimulated neutrophils with Phorbol 12*-*myristate 13-acetate (PMA, 20 nM).

### Effect of MPO Inhibitor or NADPH Oxidase Inhibitor or Hydrogen Peroxide (H_2_O_2_) on Ft-Induced NETs

*Ft* infection has been shown to inhibit ROS generation by inhibiting NADPH oxidase activity within 60 min (15). We previously demonstrated that NETs generation is induced by redox enzymes such as MPO and superoxide dismutase (SOD) (19). To determine the role of MPO in *Ft*-infected neutrophils (1:20 MOI) were incubated with or without a MPO inhibitor 4-aminobenzoic acid hydrazide (4-ABAH, 100 μM) or H_2_O_2_, a MPO substrate (180 μM). NETs release was observed after 4 h. *Ft*-mediated NADPH oxidase inhibition was reported until 60 min after infection (15). Since NETs were observed after 4 h and to test whether *Ft*-induced NETs release is independent of NADPH oxidase activity, *Ft*-infected neutrophils were incubated in the presence of Diphenyleneiodonium (DPI, 10 μM) to completely block NADPH oxidase activity and the release of NETs evaluated after 4 h. PMA at 20 nM concentration was used as a positive control for NETs induction. These experiments were repeated three times. A total of 5 fields were counted for NETs positive staining and expressed as the percentage of total NETs-positive cells in different conditions.

### Microbicidal Activity of NETs

To determine if NETs have bactericidal activity against *Francisella*, mouse neutrophils were isolated as described above. Neutrophils were seeded onto poly-D-lysine coated coverslips as described above and incubated with *Francisella* at 20:1 ratio dilution. After infection, cultures were lysed with freeze/thaw cycles and 0.1% Triton-X 100 in PBS for 3 min. Neutrophil lysates were serially diluted in PBS and plated onto chocolate agar plates for bacterial counts. For control, *Francisella* seeded without neutrophils was used. Surviving colonies were counted after 72 h. Further, to examine if NETs induced during infection trap the *Ft* bacteria, neutrophils were infected with *Ft*-LVS-GFP. At 3 h post infection, cells were stained with DAPI. Bright field and green and blue fluorescence images were captured at 1000× using Olympus fluorescence microscope.

### Impact of Antibiotic Ciprofloxacin Treatment on NETosis *in vitro*

To assess impact of antibiotic treatment on NETosis, mouse neutrophils were infected with *Ft*-LVS (1:20 MOI) and incubated with ciprofloxacin (10and 20 μg/ml) prepared in sterile PBS. After 4 h, cells were stained with nuclear dye, DAPI and determined NETs release was determined as described above. To assess if ciprofloxacin has direct impact on NETosis, neutrophils were pre-incubated with 20 μg/ml ciprofloxacin for 20 min, followed by stimulation with PMA (20 nM). NETs release was evaluated after 4 h.

### Effect of Treatment Ciprofloxacin Treatment on Ft-LVS Infection in Mice

To further assess impact of ciprofloxacin on NETosis during Ft infection in mice, BALB/c mice were infected with 1000 CFU of *Ft*-LVS. Ciprofloxacin (30 mg/kg) was administered orally starting at 48 h post infection, twice daily and given for 3 days. Animals were sacrificed at 5 days post infection. Lungs were lavaged with equal volumes of PBS and NETosis was analyzed by evaluating BAL for released H2A, citH3 and PAD4 levels by western blot analysis as described above.

### Statistical Analyses

All the data were expressed as the means ± SE. Statistical analyses were performed using Student’s *t*-test or analysis of variance (ANOVA) using Excel or GraphPad Prism 7 software. A value of *p* < 0.05 was considered statistically significant.

## Results

### Ft Infection in Mice Induces Widespread NETosis and Accumulation of Extracellular Histones

**FIGURE 1:**
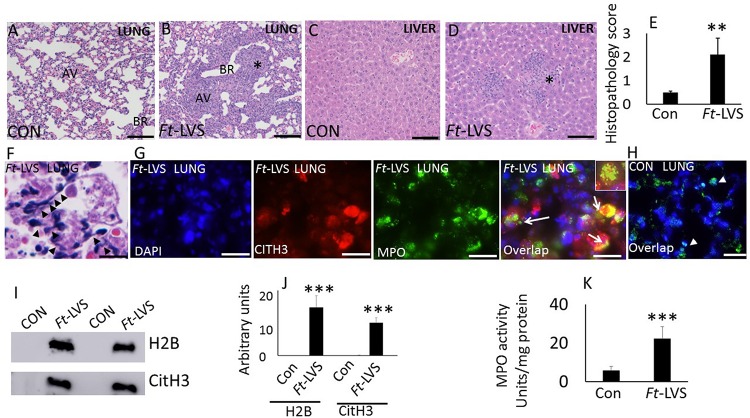
*In vivo* evidence for NETs formation during Ft infection in mice. Paraffin-embedded lung tissues from mice challenged with lethal *Ft*-LVS were stained with hematoxylin-eosin or by immunofluorescence at 5 days post infection. **(A,B)** Mock-infected and *Ft*-LVS infected mouse lungs. *Ft-LVS* infected lungs displayed nodular hemorrhagic lesions with severe alveolar destruction and dense neutrophil influx (asterisk). **(C,D)** Histopathology of mock and *Ft*-LVS infected mouse liver. Liver displays hepatic necrosis with degenerative neutrophil infiltrations (asterisk) AV- alveoli; BR-bronchioles.**(E)**Semi-quantitative scoring of lung histopathology from control and *Ft*-LVS infected mice. **(F)** Extensive induction of NETs was detected in the alveoli (black arrow heads), within severely affected areas of infected lungs. **(G,H)** Immunostaining for NETs identified by co-localization of DNA (blue) with citrullinated H3 (citH3, red) and neutrophil granule marker MPO (green). Insert shows co-localization of citH3 with MPO, indicative of NETs release by lung neutrophils. **(I)**Detection of released NETs and extracellular histones in the BAL fluids by Western blot analysis. All the western blots were carried out by loading equal volumes of BAL from control and infected animals. **(J)** Densitometry analyses of histones H2B, and citH3. Control (Con) or *Ft*-LVS infected mice. **(K)** MPO activity in lung homogenates from control and infected mice. White arrows indicate NETs formation. Results are expressed as means ± SE. *N* = 5 in each group. Student’s *t*-test was performed for comparing CitH3, H2B, and MPO densitometry values between control and *Ft*-LVS infected mice and *p* value of <0.05 was considered as significant. Scale bars: 40 μm **(A)**; 20 μm (**C–D)**. ^∗∗^ depicts *P* < 0.01, ^∗∗∗^ depicts *P* < 0.001 vs. control.

BALB/C mice were infected intranasally with 10^3^ CFU of *Ft*-LVS or PBS (for mock infection) and euthanized at 5 dpi. Histopathology and release of NETs were evaluated by H&E staining, immunohistochemistry and western blot analysis. Mock-infected mouse lungs and liver did not show any pathologic lesions (Figures 1A,C). *Ft*-infected mouse lungs displayed widespread nodular hemorrhagic lesions with massive degenerative and non-degenerative neutrophilic influx, necrotic debris and fibrin deposition (Figure 1B). The liver tissue from *Ft*-infected mice displayed mild hepatic necrosis with degenerative neutrophil infiltrations (Figure 1D). A semi-quantitative lung pathology scoring revealed significant pathology in *Ft* infected mice (Figure 1E). Intriguingly, prominent NETs release appeared individually or bundled within the areas of tissue damage, predominantly within nodular regions that were filled with neutrophils (Figure 1F). Owing to the processing and thin sectioning of fixed tissue, the release of NETs frequently appeared as elongated extensions with cellular origin or condensed small chromatin extensions from the neutrophils on H&E staining (Figure 1F). To validate the released NETs, we performed immunostaining that revealed the presence of citH3 and MPO co-localizing or appearing in the same cell depending on the status of the cells undergoing NETosis. The released NETs prominently appear within the affected regions of the lungs (Figure 1G). Prominent NETs release was observed in necrotic regions with discrete neutrophilic inflammations in the liver (Supplementary Figure S1). Mock-infected lungs did not show any staining for presence of citH3 or MPO (Figure 1H). To further validate the release of NETs in *Ft* infection, we performed western blot analysis of the BAL fluids collected from mock and *Ft* infected mice. Accumulation of histones (H2B) and modified histones (citH3, indicative of NETs release) was measured by western blot analysis (Figure 1I). Lungs were lavaged with equal volumes of PBS and the BAL densitometry analysis has shown significant accumulation of both extracellular histones and NETs in *Ft* infected lungs (Figure 1J). *Ft* infection of neutrophils has been shown to impair neutrophil activity and respiratory burst *in vitro* (15). To assess whether *Ft* infected lungs also exhibit impaired neutrophil activity *in vivo*, we measured lung MPO activities in mock and *Ft* infected mice. Interestingly, we found a fivefold increase in lung MPO activity (units per mg protein) in the lung homogenates of *Ft* infected mice (Figure 1K), thus indicating massive neutrophil influx.

### The Released NETs Entangle With Alveolar Epithelium That Displays Membrane Disruption and Alveolar Damage

**FIGURE 2:**
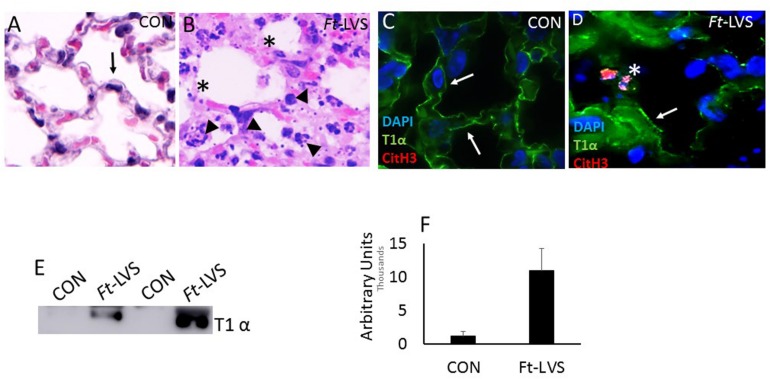
*In vivo* accumulation of NETs within the damaged lungs and evidence for alveolar epithelial injury.**(A)** Mock-infected mice displayed normal alveolar structures (black arrow). **(B)** *Ft*-LVS infected mouse lungs on H&E staining showed released NETs (arrow head) attached onto the alveolus that shows disrupted epithelium (asterisk). **(C)** Mock-infected lungs show typical continuous staining for T1α on type I alveolar epithelium (white arrow). **(D)** *Ft*-LVS infected mouse lungs displayed disrupted alveolar epithelium (asterisk) upon close interaction with NETs (citH3 positive staining, red). Other areas of alveoli show normal epithelium shown as continuous staining for T1α (white arrow). DNA (blue) with citH3 (red), and T1α (green). White arrows indicate T1α staining and asterisks indicate damaged alveolar epithelial lining. **(E)** Western blot analysis for the release of T1α by analysis in BAL fluids. All the western blots were carried out by loading equal volumes of BAL from control and infected animals. **(F)** Densitometry analysis for T1α indicates significant alveolar epithelial injury in *Ft* infection. Results are expressed as means ± SE. Student’s *t*-test was performed for comparing T1α densitometry values between control and *Ft*-LVS infected mice.*N* = 5 mice per group. Scale bars: 20 μm. ^∗∗^ depicts *P* < 0.01 vs. control.

We have demonstrated earlier that the close attachment of NETs contribute significant cytopathic effect on alveolar epithelium and endothelium in influenza and *Streptococcus pneumoniae* superinfection following influenza challenge in mice (19). *Ft* bacteria interact with both type I and type II cells and induces cytotoxicity in type II cells, but not type I cells (28). To assess whether NETs interact with alveolar epithelium and trigger alveolitis, we evaluated less damaged areas showing normal alveolar architecture by histopathology and immunostaining analysis. As shown in Figure 2A, mock-infected mouse lungs displayed normal alveoli (black arrow) and immunostaining with Type I alveolar epithelial membrane marker, T1α that appears as continuous staining (Figure 2C, white arrow) on the membrane, as 95% of alveoli are covered with alveolar type I epithelium. Upon *Ft* infection, the released NETs show widespread attachment to the alveolar epithelium (Figure 2B, arrow head), which exhibited disrupted alveoli (Figures 2B,D asterisk). The areas that are adjacent to nodular regions appear normal with intact epithelial membrane staining (arrow). To further validate alveolar epithelial damage, we evaluated western blot and immunostaining for T1 α (a membrane protein of alveolar type I cell), which was significantly increased in infected mice (Figures 2E,F). These results indicate that the released NETs could contribute to collateral alveolar injury, which may exacerbate pathophysiology in *Ft* infection.

### Ft Infected Domestic Cats and Rabbits Exhibit Severe Lung Pathology Associated With Extensive NETs Release

**FIGURE 3:**
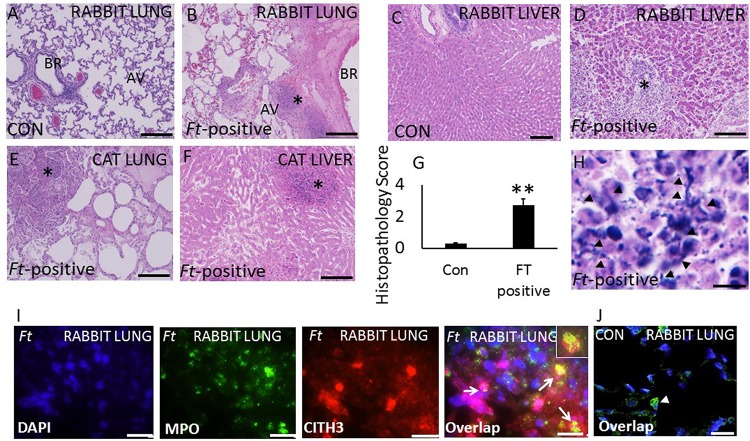
Evidence for the induction of NETosis *in* Ft-positive rabbits and cats. **(A,B)**H&E images of lung, and liver samples from control rabbits. Severe alveolar damage and neutrophil influx (asterisk). **(C,D)** *Ft*-positive rabbits hepatic degeneration and dense neutrophil influx (asterisk) and **(E,F)** *Ft*-positive cats. Degeneration and dense neutrophil influx in the cat lung and liver (asterisk). **(G)** Semi-quantitative histopathology scores of control (Con) and *Ft*-positive rabbit lungs. **(H)** Extensive induction of NETs was detected within the alveoli (black arrow heads) within severely affected areas of the rabbit lungs. **(I,J)** Immunostaining for NETs formation in infected lungs. NETs were identified by co-localization of DNA (blue) with citH3 (red) and neutrophil granule marker MPO (green). Insert shows co-localization of citH3 with MPO indication of NETs release by lung neutrophils. *N* = 4 *Ft*-positive rabbits; 3 *Ft*-positive cats and 3 uninfected rabbits. Student’s *t*-test was performed for comparing T1α densitometry values between control and *Ft*-LVS infected rabbits.Results are expressed as means ± SE. Scale bars: 20 μm. ^∗∗^*P* < 0.01 vs. control.

Severely ill domestic rabbits and cats with symptoms of high fever, lethargy, respiratory complications, diarrhea, and nasal blood discharge were brought to the Oklahoma State University veterinary clinic. Animals were euthanized, subjected to necropsy analysis, and *Ft* infection was confirmed by real-time PCR analysis of liver and spleen samples. We have included uninfected rabbit lung and live samples as controls. No pathologic lesions were observed in the lungs and liver samples collected from uninfected rabbits (Figures 3A,C). Histopathologic analysis of rabbit lungs revealed typical nodular and hemorrhagic lesions in *Ft*-positive rabbits (Figure 3B, asterisk). Necrotic foci were composed of degenerated neutrophils accompanied by cellular debris, abundant fibrin. In liver, hepatic architecture was multifocally effaced by discrete foci of coagulative necrosis of the liver (Figure 3D, asterisk). The necrotic foci consisted of dead, numerous non-degenerate and degenerate neutrophils, eosinophilic hepatocytes, and histiocytes (Kupffer cells). Three uninfected rabbits were included as controls in this study. Pathologic lesions in *Ft*-positive cats were similar to rabbits with widespread typical nodular and hemorrhagic lesions in the lungs; which often display discrete foci of necrosis and neutrophilic infiltrates (Figure 3E). The liver tissue was effaced by discrete foci of coagulative necrosis. The necrotic foci were comprised of dead, eosinophilic hepatocytes, numerous non-degenerate and degenerate neutrophils, and sparse hemorrhage and some fibrin strands (Figure 3F). Total histopathologic scores between control and *Ft*-positive rabbits are shown in Figure 3G. Intriguingly, prominent NETosis was observed in lung and liver tissue of both *Ft*-positive rabbits and cats. Histopathology analysis also showed NETs, which appeared as elongated extensions with cellular origin by H&E staining (Figure 3H). Immunostaining analysis for identification of NETs revealed prominent NETs in *Ft*-positive rabbit lungs, but not in uninfected rabbit lungs (Figure 3I). Prominent NETs appeared within the liver granulomas with discrete neutrophilic necrosis (Supplementary Figure S2) of *Ft*-positive rabbits. Control rabbit lungs did not show any staining for citH3 and MPO (Figure 3J).

### Ft Induces NETs, Which Is Dependent on MOI of Infection *in vitro*

**FIGURE 4:**
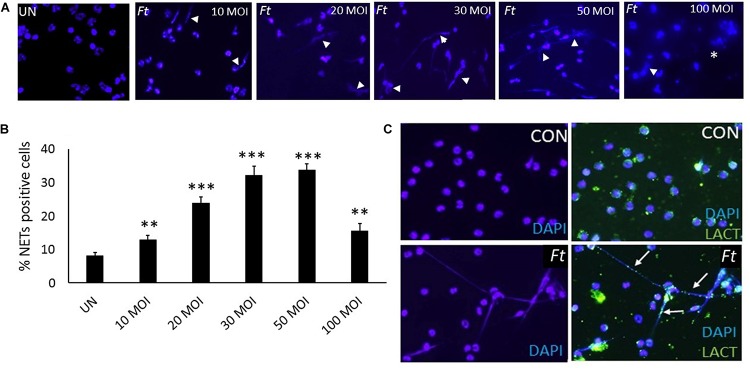
Ft-infection induces NETosis *in vitro.* **(A)**Neutrophils were infected with different MOI of the *Ft*-LVS (1:10, 1:20, 1:30, 1:50, and 1:100) and NETs induction evaluated after 4 h by staining with DAPI, a nuclear dye (white arrow heads).**(B)** Significant induction of NETs release was observed upon *Ft*-LVS infection and NETs formation increased with the increase in MOI. **(C)** NETs release was validated using lactoferrin, which appeared as “green beads on strand” staining on chromatin fibers (white arrow). The values are average of three independent experiments. Results are expressed as means ± SE. Scale bars: 20 μm. One-way ANOVA with Tukey’s multiple comparison was analyzed. ^∗∗^ depicts *P* < 0.01 and ^∗∗∗^ depicts *P* < 0.001vs. uninfected control.

**FIGURE 5:**
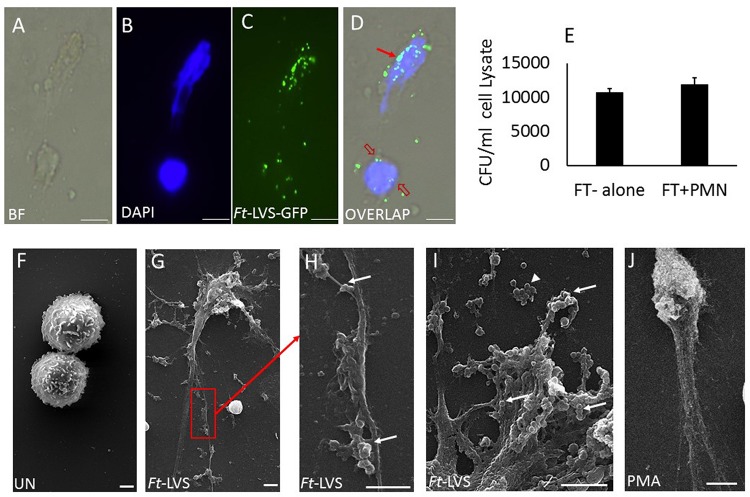
NETs trap Ft-LVS bacteria, but fails to kill. To test whether NETs trap *Ft* bacteria, murine neutrophils were infected with green fluorescence protein (GFP) labelled *Ft* bacteria, *Ft*-LVS-GFP at MOI of 1:20. **(A–D)** Immunostaining analysis clearly demonstrates trapping of*Ft*-LVS-GFP bacteria in NETs. Red arrow shows *Ft* trapped in NETs. Red open arrow shows Ft in the cytoplasm. **(E)** However, no differences in CFU were found in the presence of NETs. **(F–I)** Scanning electron microscopy of NETs formed during Ft infection. **(J)** Positive control of NETs induction when neutrophils were stimulated with PMA. BF–bright field image. White arrow – bacteria attached to NETs. Arrow head – cluster of free bacteria not attached to NETs. The values are average of three independent experiments. Scale Bar for **A–D** = 20 μm; Scale Bar for **F–J** = 1 μm. Results are expressed as means ± SE.

Mouse neutrophils were infected with different MOI (1:10, 1:20, 1:30, 1:50, and 1:100) of *Ft* for 4 h. The released NETs were identified by staining with DAPI (nuclear dye) to identify NETs. *Ft* infection induced NETs release by 4 h (Figure 4A) and NETs induction was enhanced with the increase in MOI of *Ft* infection (Figure 4B). However, at MOI of 1: 100 significant number of neutrophils exhibited disintegrated nuclei (Figure 4A, asterisk), but decrease in NETs release. The released NETs were confirmed by immunostaining analysis using a NETs marker, lactoferrin, which appeared as “beads on a string” staining on released NETs (Figure 4C). We have earlier found that released NETs trap bacteria in the extracellular environment (19, 29). To assess if NETs released during *Ft* infection interact with bacteria, we infected neutrophils with *Ft*-LVS-GFP, i.e. GFP-labeled bacteria. As shown in Figures 5A–D, the ingested bacteria were trapped in the NETs releasing from the neutrophils and bacteria were also present in the cytoplasm of the infected cell. These studies indicate that NETs occur during active ingestion of the bacteria. Although NETs appeared to trap the bacteria, we did not find bactericidal effects of NETs (Figure 5E). To further validate these results, we have performed scanning electron microscopy to identify *Ft-LVS* interaction with released NETs. We observed clusters or single bacterial cell attached to the NETs chromatin strands or trapped in NETs (Figures 5F–I). We have also included SEM of NETs released by neutrophils stimulated with Phorbol 12-myristate 13-acetate (PMA) (Figure 5J).

### Active Bacterial Infection Required for NETs Formation

**FIGURE 6:**
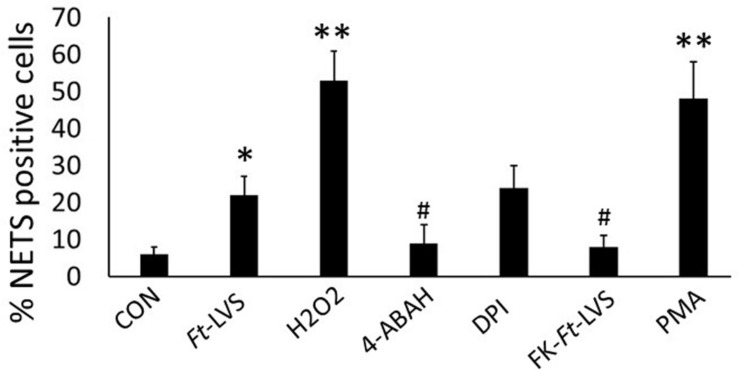
Ft induced NETosis is dependent on MPO activity. Neutrophils were infected with *Ft*-LVS (1:20 MOI) and NETs induction evaluated by immunostaining for lactoferrin and counter stained with DAPI. *Ft*-induced NETosis require active bacterial infection. To test this, *Ft*-LVS was killed with formalin. Formalin killed (FK), FK-*Ft*-LVS fail to induce NETosis. Activation of MPO with its substrate, H_2_O_2_ significantly enhanced NETs release, while pharmacological blocking of MPO activity with 4-ABAH significantly inhibited *Ft*-LVS induced NETosis. Similarly, blocking NADPH oxidase with DPI did not show any difference compared to neutrophils infected with *Ft*-LVS alone infected neutrophils. PMA stimulation was used as positive control. One-way ANOVA with Tukey’s multiple comparison was analyzed. ^∗^*P* < 0.05 vs. control; ^#^*P* < 0.05 and ^∗∗^*P* < 0.001 vs.*Ft*-LVS.

Earlier studies have shown that live bacterial infection is required for inhibition of phagocytic activity and formalin killed bacteria fails to prolong neutrophil life span or to delay apoptotic death in neutrophils. To assess whether live bacterial infection is essential for induction of NETosis, formalin killed *Ft*-LVS was incubated with neutrophils. The formalin-killed bacteria (FK-*Ft*-LVS) did not induce NETs, thus suggesting that NETosis is an active mechanism that requires bacterial phagocytosis by neutrophils. NETs induction by PMA showed significant induction of NETosis (Figure 6).

### Ft-Induced NETs Is Dependent on MPO Activity

NADPH oxidase activity is required for generation of ROS during NETosis. Since *Ft* inhibits NADPH oxidase activity, we hypothesized a possibility for NADPH oxidase-independent NETs release during *Ft* infection. MPO and neutrophil elastase released from azurophilic granules anchor chromatin scaffolds in NETs, and mediate histone degradation during NETs formation (30, 31). Since we found significant induction of MPO activity in *Ft*-infected lungs, we asked whether MPO is critical in *Ft*-induced NETs formation. To test this, mouse neutrophils were infected with *Ft* in the presence or absence of pharmacological blocker of MPO (4-ABAH). Blocking with 4-ABAH significantly inhibited NETs release (Figure 6). To assess if induction of MPO activity potentiates NETosis, *Ft* infected neutrophils were incubated in the presence of a MPO substrate, H_2_O_2_. Interestingly, the addition of H_2_O_2_ significantly increased NETs release, thus indicating that activation of MPO potentiates NETs generation. To further assess NADPH oxidase independence in *Ft* induced NETs, neutrophils were incubated in the presence of NADPH oxidase inhibitor to completely abolish the NADPH oxidase activity. The addition of NADPH oxidase inhibitor also induces NETs release similar to *Ft*-alone group, suggesting that *Ft*-induced NETosis is NADPH oxidase independent. These findings indicate that MPO activity is critical in the release of NETs during *Ft* infection.

### Effect of Antibiotic Ciprofloxacin on NETosis *in vivo* and *in vitro*

**FIGURE 7:**
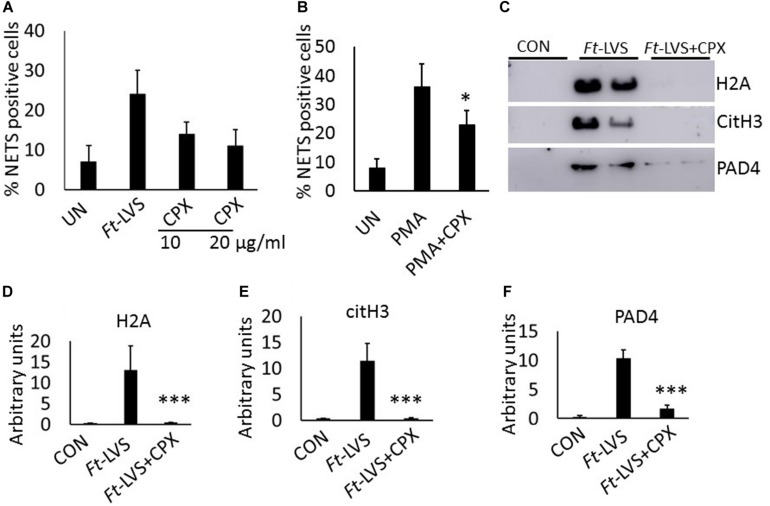
Impact of ciprofloxacin on NETosis *in vitro* and *in vivo*. **(A)** Murine neutrophils were infected with *Ft*-LVS at 1:20 MOI in the presence of ciprofloxacin (10 and 20 μg/ml). The release of NETs was determined. **(B)** Effect of ciprofloxacin on PMA-induced NETosis. **(C)** Western blot analysis for the release of H2A, citH3, and PAD4 in BAL fluids of infected (10^3^ CFU of *Ft*-LVS) and ciprofloxacin (30 mg/kg) treated BALB/c mice at 5 days post infection. All the western blots were carried out by loading equal volumes of BAL from control and infected animals. **(D–F)** Densitometry analysis of H2A, citH3 and PAD4 (*n* = 4). **(A,B)** The values are average of three independent experiments. Results are expressed as means ± SE. Student’s *t*-test was performed for comparing T1α densitometry values between control and *Ft*-LVS infected mice and *p* value of <0.05 was considered as significant. ^∗^*P* < 0.05, vs.*Ft*-LVS or PMA, ^∗∗∗^*P* < 0.001 vs.*Ft*-LVS.

To assess impact of antibiotic treatment on NETosis, *Ft* infected neutrophils were incubated with different concentrations of ciprofloxacin. Significant decrease in NETs formation was observed compared to *Ft* infection (Figure 7A). To determine if ciprofloxacin has direct impact on NETosis, neutrophils were incubated with ciprofloxacin and stimulated with PMA. Ciprofloxacin incubation reduced PMA-induced NETs (Figure 7B). To further assess impact of ciprofloxacin on NETosis during *Ft* infection in mice, infected mice were treated with ciprofloxacin and analyzed for NETs release by western blot analysis. The antibiotic significantly abrogated NETs induction and release of extracellular histones, as evident by decreased H2A and citH3 levels (Figures 7C–E). Further ciprofloxacin treatment also reduced lung PAD4 levels (Figures 7C,F), thus indicating that antibiotic treatment significantly suppressed NETosis *in vivo*.

## Discussion

Our findings demonstrate that *Ft* infection triggers NETosis both *in vivo* and *in vitro*. The released NETs prominently appeared in the lungs and liver of *Ft*-LVS infected mice, and in clinically diagnosed *Ft*-positive domestic cats and rabbits. Although aggravated neutrophil influx has been associated with in host tissue injury in severe *Ft* infections, their role in *Ft* pathophysiology is not completely understood. Our findings reveal that neutrophils recruited at infection site exhibit high MPO activity and undergo NETopathic cell death during active *Ft* infection. The released chromatin strands released from NETs prominently appear within nodular hemorrhagic lesions, indicating a potential role of NETs in aggravating collateral tissue damage. We found that *Ft-*LVS infection in neutrophils induces NETs release *in vitro*. Further, *Ft-*LVS-induced NETs release required MPO activity and was independent of NADPH oxidase activity. Antibiotic treatment with ciprofloxacin significantly suppressed NETosis both *in vivo* and *in vitro*. These studies provide a novel basis for the role of NETs in *Ft* pathophysiology.

Previous studies have shown that *Ft* parasitizes inside the neutrophil and suppresses the neutrophil’s antimicrobial defense by impairing respiratory burst, thus allowing the *Ft* to escape from phagosomal killing (15–18).*Ft*-mediated suppression of neutrophil respiratory burst occurs *via* disruption of NADPH oxidase enzyme complex that aids in bacterial survival in these immune cells (15, 32). Simultaneously, *Ft* also enhances neutrophil life-span by inhibiting their apoptotic signaling pathway *in vitro* (18, 33, 34). Although studies on *Ft-*neutrophil interactions implicate how *Ft* can suppresses host innate immune defense and neutrophil functionality *in vitro*, the fate of massive neutrophils that are recruited at the infection site remain poorly defined. Furthermore, activation status of these infiltrated neutrophils are so far unknown. Our studies have identified that lung-sequestered neutrophils are functionally active and generate NETs. We found strong immunostaining of NETs components (including citH3 and MPO) in the damaged lungs and liver of mice infected with *Ft-*LVS as well as *Ft*-positive domestic rabbits and cats. It is also noted that only a subpopulation of neutrophils appeared to release NETs, since we also detected MPO positive neutrophils without citH3 staining with disintegrated nuclei that were possibly undergoing apoptosis or necrosis.

Neutrophil extracellular traps are formed as large extracellular web-like chromatin strands that were initially proposed as a defense mechanism against invading pathogens (24). However, excessive release of NETs aggravate tissue injury and death as reported in several clinical and disease conditions (20–22, 35, 36). Although aerosol route of *Ft* infection causes severe pneumonia tularemia pneumonia with high fatalities, the pathophysiology in this devastating respiratory disease is not yet completely understood (37). Careful examination of the *Ft-*infected mouse, domestic rabbit and cat lungs revealed that the NETs DNA fibers were frequently entangled to the alveolar epithelium, suggesting suggests that these structures may contribute to damage of thin alveolar-capillary barrier. There are several possibilities how these NETs could aggravate *Ft* pathophysiology. First, the close-proximity of the NETs fibers carrying toxic nuclear and granule proteins such as histones, MPO, MMPs, and neutrophil elastase could disrupt thin alveolar-capillary barrier, thereby enhancing the systemic spread of the bacteria (38). In support of this, we recently showed that extracellular histones induce cytotoxic response in alveolar epithelial cells *in vitro* and *in vivo*. Interestingly, despite presence of excessive NETs in dual-infected mice (*Streptococcus pneumoniae* superinfection, following influenza), aggravated alveolar disruption and augmented systemic spread of the bacteria were noted, thus suggesting that NETs-mediated injury could compromise alveolar function (19). Second, *Ft* infection of alveolar type II epithelial cells and alveolar macrophages triggers high induction of cytokines including IL-8, MCP-1, and IL-1β(28, 39). These chemoattractant cytokines potentially modulate inflammatory status in the lungs by attracting massive neutrophil influx and activation at the infection site. In addition, we report here that NETs released during *Ft* infection, entangle alveolar epithelial cells and disrupt thin alveolar epithelial bed. Indeed, the DNA fibers entangled with alveolar epithelium has been implicated in the pathophysiology of influenza, bacterial pneumonia and sepsis (19, 25, 40). These findings suggest that a cumulative effect of *Ft*-inflicted alveolar type II epithelial injury together with NETs-mediated alveolitis could contribute to widespread pathophysiology in severe *Ft* infections. Work is currently underway to further characterize mechanisms of *Ft*-induced NETs.

Next, to validate whether *Ft*-neutrophil interaction triggers NETosis, we infected neutrophils with different MOI of *Ft*-LVS. Our data has shown that *Ft* infection induces NETs release, which is dependent on MOI. The lack of NETs formation with formalin-killed *Ft* bacteria suggested that active bacterial infection is required for the formation of NETs. The mechanism of NETosis is a complex process and mediated by various factors. Previously, it has been shown that *Ft* infection disrupts assembly of gp91/p22*phox* or p47/p67*phox* domains in NADPH oxidase, and thereby impairs neutrophil respiratory burst and activity observed within 60 min after infection (15). Although activity of NADPH oxidase has been linked to NETs formation, it is not indispensible in forming the NETs as NADPH oxidase independent NETosis has been identified in *Staphylococcus aureus* infection (41). Moreover, NETs release was observed 4 h after *Ft* infection and NADPH oxidase activity during that time is unknown. To exclude the involvement of NADPH oxidase activity, *Ft*-infected neutrophils were inhibited by pharmacological drug against NADPH oxidase. Interestingly, blocking NADPH oxidase did not show any impact on NETs release indicating that *Ft* induced NETs release occurred independently of NADPH oxidase activity. Our studies have revealed that pharmacological blocking of MPO significantly reduced NETs generation, whereas induction of MPO activity by addition of H2O2 potentiated NETs release. Further studies are needed to determine whether *Ft* infection induces MPO expression or enhances its activity both *in vitro* and *in vivo*. Taken together these findings suggest that recruited neutrophils into the infection site potentially induce NETosis, which is dependent on MPO activity.

Antibiotic therapy is an effective treatment choice for *Ft* infections(42). The use of gentamycin and tetracyclins are effective *Ft*-treatments. However, some clinical reports have documented failures with relapse when these antibiotics were used (43, 44). On the other hand, gentamicin also inhibits neutrophil chemotaxis function (45). Although ciprofloxacin and doxycycline are recommended for milder forms of the disease, treatment with doxycycline is contraindicated in children under 8 years of age (46). These findings indicate that a better understanding of therapeutic effectiveness in antibiotic treatments and their association with host response is needed. The involvement of host-related pathogenesis in *Ft* infection is still not completely understood. Our studies indicate a possible mechanism how the excessive neutrophils could exacerbate pulmonary pathology in *Ft* infections. With our findings indicating extensive release of NETs and their pathogenic link in *Ft*-infection, we assessed the impact of antibiotic treatment on NETs. Interestingly, antibiotic treatment suppressed NETosis both *in vitro* and *in vivo* and we found direct inhibitory effect of ciprofloxacin in PMA-induced NETs release, suggesting a direct suppression of NETs formation by antibiotic treatment. This was further evident by decreased citH3 and PAD4 levels in infected lungs. One of the prominent characteristics of the NETs is to trap and kill the microbial pathogen in the extracellular environment. Interestingly, neutrophils infected with GFP-labeled *Ft* bacteria displayed a significant number of bacteria being trapped in the NETs chromatin fibers. However, no bactericidal effects were observed, thus suggesting that NETs induced during *Ft* infection can contribute to lung injury, but have limited bactericidal activity.

Taken together, these studies indicate that massive induction of NETs could significantly contribute to acute pulmonary damage in *Ft*-infection. Further, we found that massive neutrophils recruited at the infection site are functionally active and undergo NETosis. The released NETs attach and disrupt alveolar epithelial bed. We also found that *Ft-*infection induces NETs release *in vitro*. Furthermore, we document that NETs formation is an active process and is dependent on MPO activity. Hence, intervention strategies to inhibit NETosis or targeting MPO may have potential therapeutic impact to ameliorate the severe pulmonary pathology in severe *Ft* infections.

## Data Availability Statement

All datasets generated for this study are included in the article/Supplementary Material.

## Ethics Statement

The animal study was reviewed and approved by the Institutional Animal Care and Use Committee (IACUC) of Oklahoma State University (protocol number VM-17-36).

## Author Contributions

SP, AR, JM, and NT contributed to conception and design of experiments. SP, MP, and NT acquired and analyzed the data. RG and TS contributed to histopathology analyses. NT, JM, and VC drafted and critically reviewed the manuscript.

## Conflict of Interest

The authors declare that the research was conducted in the absence of any commercial or financial relationships that could be construed as a potential conflict of interest.

## Supplementary Material

FIGURE S1 Immunostaining for NETs formation in infected mouse liver. NETs were identified by co-localization of DNA (blue) with citH3 (red) and neutrophil granule marker MPO (green).

FIGURE S2 Immunostaining for NETs formation in infected rabbit liver. NETs were identified by co-localization of DNA (blue) with citH3 (red) and neutrophil granule marker MPO (green).
