# Supplementary figures and images for: Production of Neutrophil Extracellular Traps Contributes to the Pathogenesis of Francisella tularemia
Source: Front Immunol. 2020 Apr 24;11:679. doi: 10.3389/fimmu.2020.00679 (PMC7193117; doi:10.3389/fimmu.2020.00679)

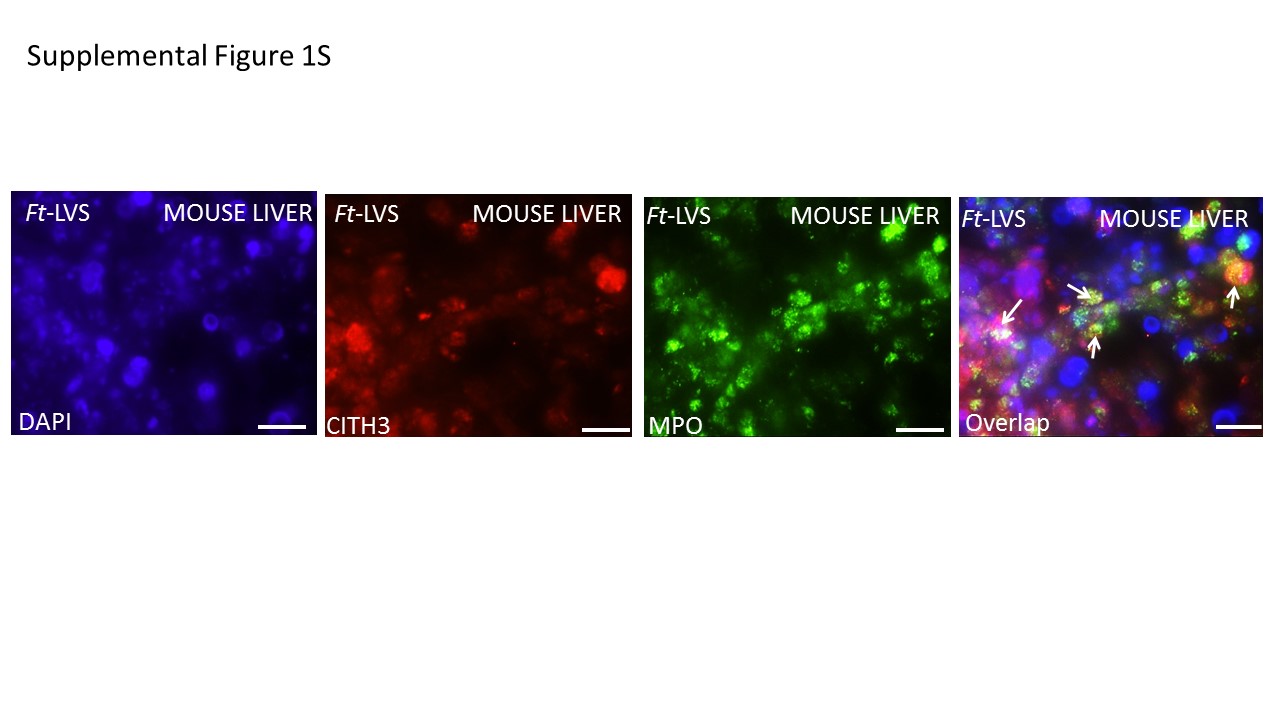

Supplement: FIGURE S1 — Immunostaining for NETs formation in infected mouse liver. NETs were identified by co-localization of DNA (blue) with citH3 (red) and neutrophil granule marker MPO (green). [file Image_1.JPEG]

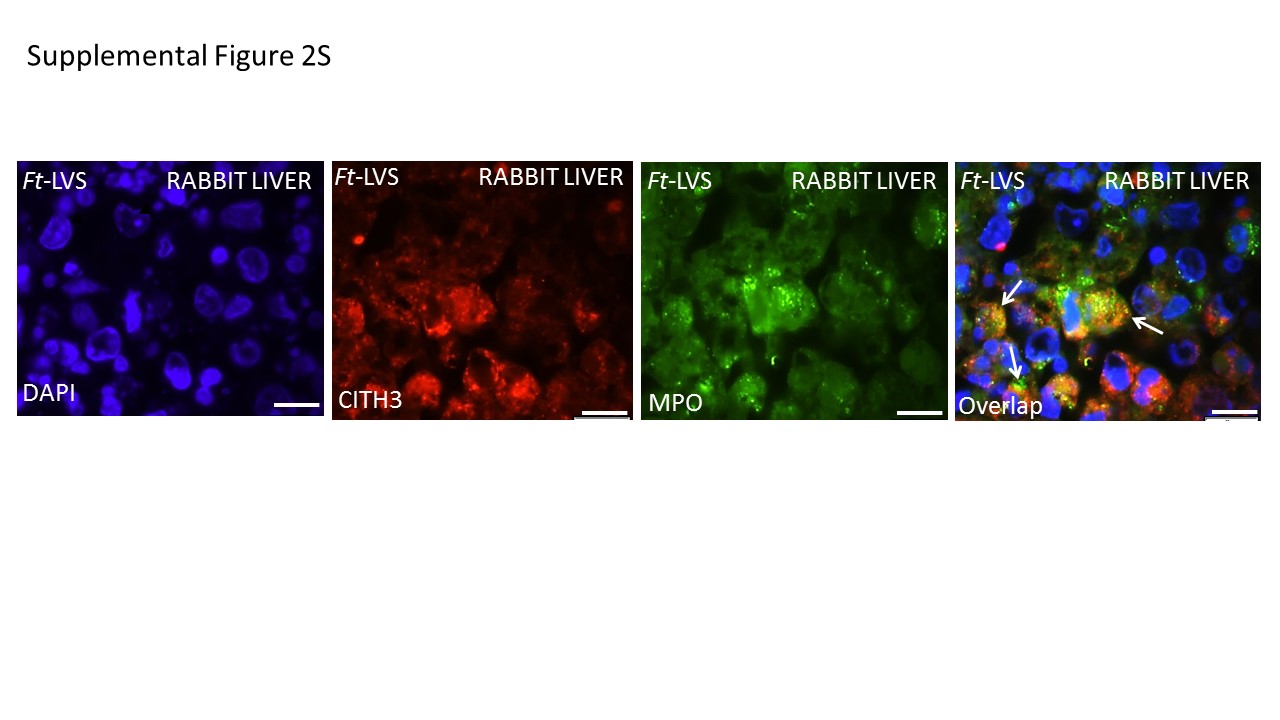

Supplement: FIGURE S2 — Immunostaining for NETs formation in infected rabbit liver. NETs were identified by co-localization of DNA (blue) with citH3 (red) and neutrophil granule marker MPO (green). [file Image_2.JPEG]
